# Construction and Analysis of Functional Networks in the Gut Microbiome of Type 2 Diabetes Patients

**DOI:** 10.1016/j.gpb.2016.02.005

**Published:** 2016-10-14

**Authors:** Lianshuo Li, Zicheng Wang, Peng He, Shining Ma, Jie Du, Rui Jiang

**Affiliations:** 1 MOE Key Laboratory of Bioinformatics and Bioinformatics Division, TNLIST/Department of Automation, Tsinghua University, Beijing 100084, China; 2 Beijing Anzhen Hospital, Capital Medical University, Beijing Institute of Heart, Lung, and Blood Vessel Diseases, Beijing 100029, China

**Keywords:** Functional network, Microbiome, Type 2 diabetes, Metagenomics, Network motif

## Abstract

Although networks of microbial species have been widely used in the analysis of 16S rRNA sequencing data of a **microbiome**, the construction and analysis of a complete microbial gene network are in general problematic because of the large number of microbial genes in **metagenomics** studies. To overcome this limitation, we propose to map microbial genes to functional units, including KEGG orthologous groups and the evolutionary genealogy of genes: Non-supervised Orthologous Groups (eggNOG) orthologous groups, to enable the construction and analysis of a microbial **functional network**. We devised two statistical methods to infer pairwise relationships between microbial functional units based on a deep sequencing dataset of gut **microbiome** from **type 2 diabetes** (T2D) patients as well as healthy controls. Networks containing such functional units and their significant interactions were constructed subsequently. We conducted a variety of analyses of global properties, local properties, and functional modules in the resulting functional networks. Our data indicate that besides the observations consistent with the current knowledge, this study provides novel biological insights into the gut **microbiome** associated with T2D.

## Introduction

Advancement of the next-generation sequencing technology has made it possible to sequence all genetic materials of a microbiome and to assemble millions of microbial genes from the data, resulting in the recent explosion of large-scale metagenomic studies in soil [1], [2], [3], air [4], [5], marine environments [6], [7], [8], and humans [9], [10], [11], as well as in many other fields [12]. As a simple and low-cost approach, sequencing of the 16S rRNA gene in whole or its hypervariable regions selectively can be employed to profile the taxonomic composition of a microbiome. This approach, together with the powerful network analysis methodology [13], has a variety of applications, such as identification of co-occurrence networks of microbial species in soil, marine environments [14], and, more recently, of humans [15], [16], [17]. The recently-proposed Boolean implication networks involving marine microbial species and environmental factors have also been reported [13], further enhancing the explanatory power of co-occurrence networks. Nevertheless, 16S rRNA sequencing can hardly be used to assess functions of a microbiome. In order to study functions of a microbial community, it is necessary to know which genes are in a community and how these genes interact with one another to support a complicated biological function.

Analogous to the available studies involving 16S rRNA analysis, construction of a co-occurrence network of all microbial genes is desirable. Nevertheless, a microbial community typically includes millions of microbial genes from tens of thousands of species. The construction of a network from such a large number of genes is nearly impossible in most metagenomic studies. On the other hand, many microbial genes are similar in sequence, suggesting that they may actually perform similar functions in a community. Therefore, it is possible to first define a set of functional units that can be mapped to known gene functions and then to study the relations between these functional units in a microbiome. Since the mapping of microbial genes to functional units is many-to-one, the number of functional units is expected to be much smaller than that of microbial genes. Moreover, interactions between such functional units can also reflect the interaction between the microbial genes, which helps to address the question how microbial genes work together to support particular functions of a microbiome.

We thus propose in this paper a framework for constructing a network of functional units in a microbiome. We define a node in such a microbial functional network as a KEGG orthologous group (KO) [18] or an evolutionary genealogy of genes: Non-supervised Orthologous Groups (eggNOG) orthologous group (OG) [19], and introduce an edge connecting a pair of nodes if the nodes show a certain correlation according to their abundance levels. Specifically, using a deep sequencing dataset of the gut microbiome from type 2 diabetes (T2D) patients [10] as a demonstration, we first map raw reads to known microbial genes and obtain abundance of a functional unit as the summation of abundance of all genes belonging to the unit. Then, we design two statistical methods to infer pairwise relationships between microbial species. Using Pearson’s correlation coefficient (PCC), we characterize the strength of an association between two functional units as the correlation of the relative abundance of the units across a number of individuals. Using a machine learning method called random forest (RF), we predict the abundance of a functional unit based on that of all the other units. Then the relative importance of certain units in this learning procedure is employed to measure the strength of an association between the response unit and the predictor unit. After filtering out weak associations between nodes, we obtain a network of functional units. Finally, we analyze global properties, local properties, and functional modules in the constructed functional networks.

## Results

### Construction of functional networks in the human gut microbiome

**Figure 1:**
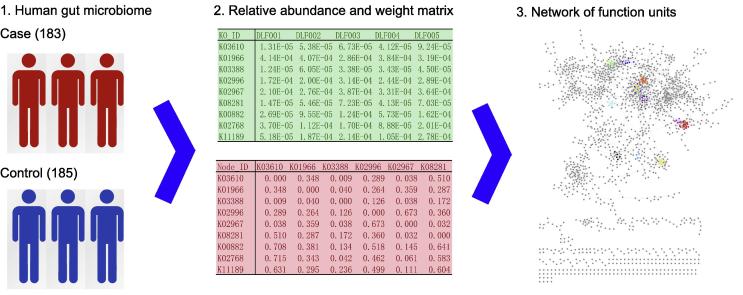
**Workflow of the proposed study** Taking raw sequencing reads of the human gut microbiome of 183 T2D patients and 185 healthy individuals as input, we adopted two statistical methods, *i.e.*, PCC and RF, to construct networks of functional units with either KO or OG, as output. To accomplish this objective in a flexible way, we divide the workflow into three independent modules: an input module for calculating relative abundance levels of the units, a network construction module for inferring pairwise relationships between functional units, and an output module for saving and visualizing the constructed networks. PCC, Pearson’s correlation coefficient; RF, random forest; KO, KEGG orthologous group; OG, evolutionary genealogy of genes: Non-supervised Orthologous Groups (eggNOG) orthologous group.

The workflow of our method is illustrated in Figure 1. Taking raw sequencing reads of the human gut microbiome of 183 T2D patients and 185 healthy individuals [10] as input, we adopted two statistical methods, *i.e.*, PCC and RF, to construct networks of functional units with either KO or OG, as output. To accomplish this objective in a flexible way, we divided the workflow into three independent modules: (1) an input module for calculating relative abundance levels of the units, (2) a network construction module for inferring pairwise relationships between functional units, and (3) an output module for saving and visualizing the constructed networks.

In the input module, we first mapped raw sequencing reads to known microbial genes (or contigs) and obtained raw read counts of the genes. We then normalized the read count of a particular gene to its length to obtain the abundance level of the gene. Using the total abundance normalization method, we divided the abundance of a particular gene by the summation of abundance levels of all genes to determine relative abundance of the gene. We finally mapped microbial genes to functional units and determined the relative abundance of a unit as the summation of abundance levels of all the genes belonging to the unit. In the network construction module, we designed two statistical methods to infer pairwise relationships between microbial species. We first quantified relationships between two functional units by means of PCC between the two vectors corresponding to the relative abundance levels of these units across a number of individuals. We then employed RF to predict the abundance of a functional unit using those of all the remaining units. Afterward, we used the relative importance of certain units in this learning procedure to measure the strength of an association between the response unit and the predictor unit. Finally, we enhanced the output module with basic statistics of the constructed network in addition to a plain text file that describes connections between functional units. Details of these methods are given in the Methods section.

### Topological properties of functional networks

We conducted a comparative analysis of the constructed functional networks of the human gut microbiome. Using KO and OG as functional units, we obtained four functional networks: KO network based on PCC, KO network based on RF, OG network based on PCC, and OG network based on RF.

**Figure 2:**
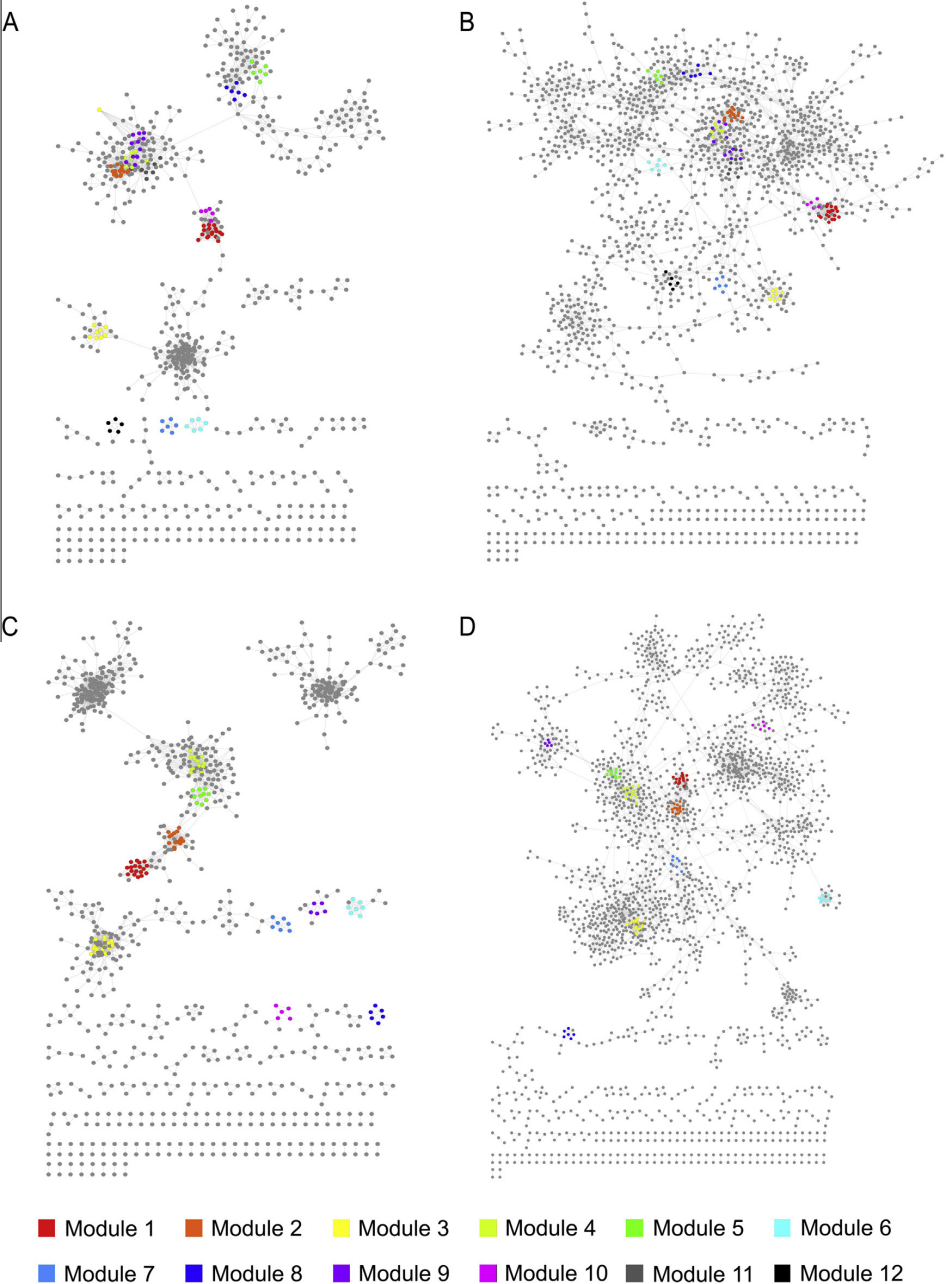
**Functional networks in human gut microbiome** **A.** PCC-based KO network. **B.** RF-based KO network. Modules were detected by using MCODE in cytoscape. Each color represents a module with the color code as follows: Module 1 (red), Module 2 (orange), Module 3 (yellow), Module 4 (light green), Module 5 (green), Module 6 (cyan), Module 7 (blue), Module 8 (dark blue), Module 9 (purple), Module 10 (pink), Module 11 (dark gray), and Module 12 (black). Common modules shared in both KO networks are indicated with same color. **C.** PCC-based OG network. **D.** RF-based OG network. Modules were detected by using MCODE in cytoscape. Each color represents a module with the color code as follows: Module 1 (red), Module 2 (orange), Module 3 (yellow), Module 4 (light green), Module 5 (green), Module 6 (cyan), Module 7 (blue), Module 8 (dark blue), Module 9 (purple), and Module 10 (pink). Common modules shared in both RF networks are indicated with same color.

As shown in Figure 2 and Table 1, we found that the KO and OG networks based on PCC share similar topological structure (Figure 2A and 2C), and so do the two networks based on RF (Figure 2B and 2D), while networks constructed using different methods vary significantly. At the default cutoff value of 1.5 for filtering weak associations between nodes in a network (see Methods), both KO networks contain 3880 edges. Nevertheless, the PCC-based KO network has only 724 nodes (Figure 2A), whereas RF-based KO network has 1687 nodes (Figure 2B), indicating that edges are distributed to nodes more uniformly when using RF in comparison with PCC. For the same reason, the PCC-based KO network has higher density and a smaller diameter than does RF-based KO network (Figure 2A and 2B). Therefore, the PCC-based KO network shows higher modularity, *i.e.*, nodes in the network tend to form denser modules; this property is also reflected by higher centralization and a greater clustering coefficient. The scale-free property is an important feature of complex networks, which requires that the degree distribution of a network fit a power law model [20]. The *R*^2^ fitting of the two KO networks is 0.830 and 0.925, respectively. Thus both networks have good scale-free properties, although the RF-based KO network performs better.

The topological properties of the OG networks (Figure 2C and 2D) are very similar to those of the KO networks based on the same method.

By comparing the performance of these two network construction methods, it is found that the PCC method tends to build a network with fewer nodes but more modules, whereas the RF method tends to build a sparser network with more nodes, a greater diameter, and good scale-free property. The two methods exhibit different characteristics and advantages, and provide useful information from different aspects. In this study, we analyze the networks based on both methods and focus on their common features.

### Highly-connected modules in functional networks

Next, to detect dense modules in the KO networks and OG networks and to explore the biological patterns in them, we identified highly-connected modules in the functional networks. A highly-connected module in a network is defined as a set of nodes where most nodes are connected with one another, *i.e.*, cliques or near-cliques. Nodes in such a module tend to be functionally similar or to interact closely thus forming a unit of a certain biological function. We applied the tool MCODE [21], a plugin for Cytoscape [22], to analyze the networks. As a result, we uncovered 12 modules present in both KO networks (Table S1) and 10 modules present in both OG networks.

**Figure 3:**
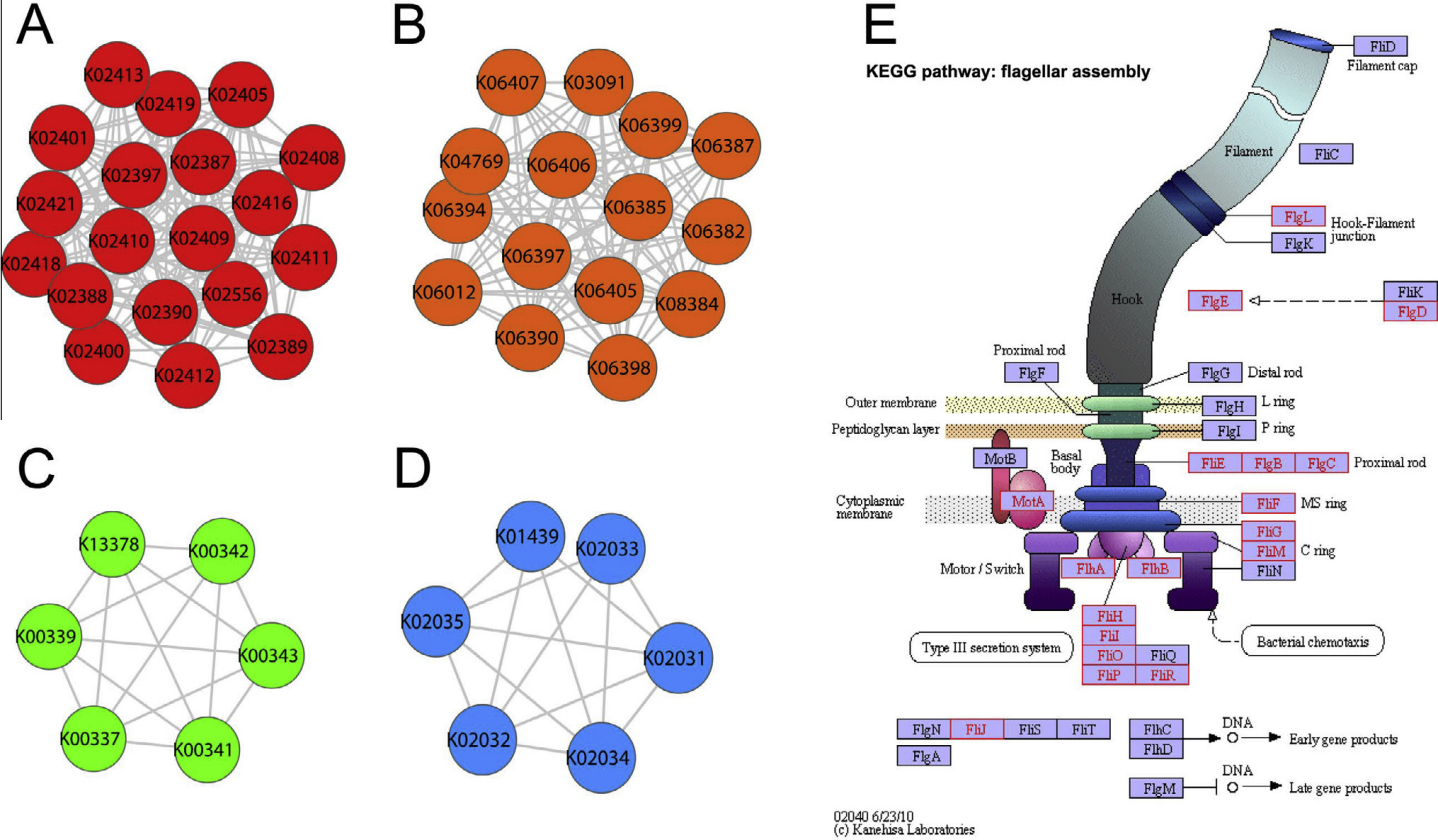
**Highly-connected clusters in functional KO networks** The two largest highly-connected clusters present in both KO networks (Module 1 and Module 2) are shown in red (**A**) and orange (**B**), respectively, whereas two smaller highly-connected clusters present in both KO networks (Module 5 and Module 7) are shown in green (**C**) and blue (**D**), respectively. Nodes of Module 1 are enriched in the KEGG pathway “Flagella assembly” (**E**).

Figure 3 shows four highly-connected modules present in both KO networks constructed by the two different methods. The largest module in KO networks (Module 1) is depicted in Figure 3A, which contains 19 nodes connected by 163 edges. Among them, 18 nodes belong to a KEGG pathway called “flagella assembly” as shown in Figure 3E. Nodes in this module represent KOs corresponding to different flagella biosynthetic proteins, and the interactions among them in the environment of the human intestines are quite strong. Consequently, this module can be detected by using both network construction methods. Figure 3B depicts the module ranked in the second place (Module 2), which contains 15 nodes and 102 edges. All KOs in this module represent proteins related to sporulation. The smaller module in Figure 3C (Module 5) is a 6-node clique, in which each KO corresponds to a protein as a subunit of the enzyme H^+^-transporting two-sector ATPase. Figure 3D shows a 6-node near-clique (Module 7) located outside the large connected components of the KO networks, and KOs in this module represent different subunits of another enzyme, NADH: ubiquinone reductase (H^+^-translocating).

Highly-connected modules detected in OG networks are similar to those in KO networks to some extent (data not shown). For example, the largest module present in both OG networks comprises 18 nodes and 134 edges. It is of note that most of the nodes correspond to functions associated with flagella, just as in KO networks.

These highly-connected modules in KO and OG networks and their biological annotations reveal that the edges in the KO and OG networks can represent interactions between proteins corresponding to the nodes, and modules can represent protein complexes with certain functions. On the other hand, these biological explanations support that the KO and OG networks are meaningful and can reflect actual interactions occurring in the human gut microbiome.

### T2D-associated markers in functional networks

Disease-associated biomarkers are of great value for the diagnosis of various human diseases and for the understanding of their pathogenesis. Via a metagenome-wide association study of the human gut microbiome, we retrieved 1345 KO and 5612 OG markers associated with T2D. Among them, 876 KO markers are T2D-enriched markers, which show significantly higher abundance in T2D samples than in control samples (one-sided Wilcoxon rank sum test, *P* < 10^−4^). On the other hand, 469 KO markers are T2D-depleted markers, which are enriched in control samples (one-sided Wilcoxon rank sum test, *P* < 10^−4^). The numbers of T2D-enriched OG markers and T2D-depleted OG markers are 3841 and 1771, respectively.

**Figure 4:**
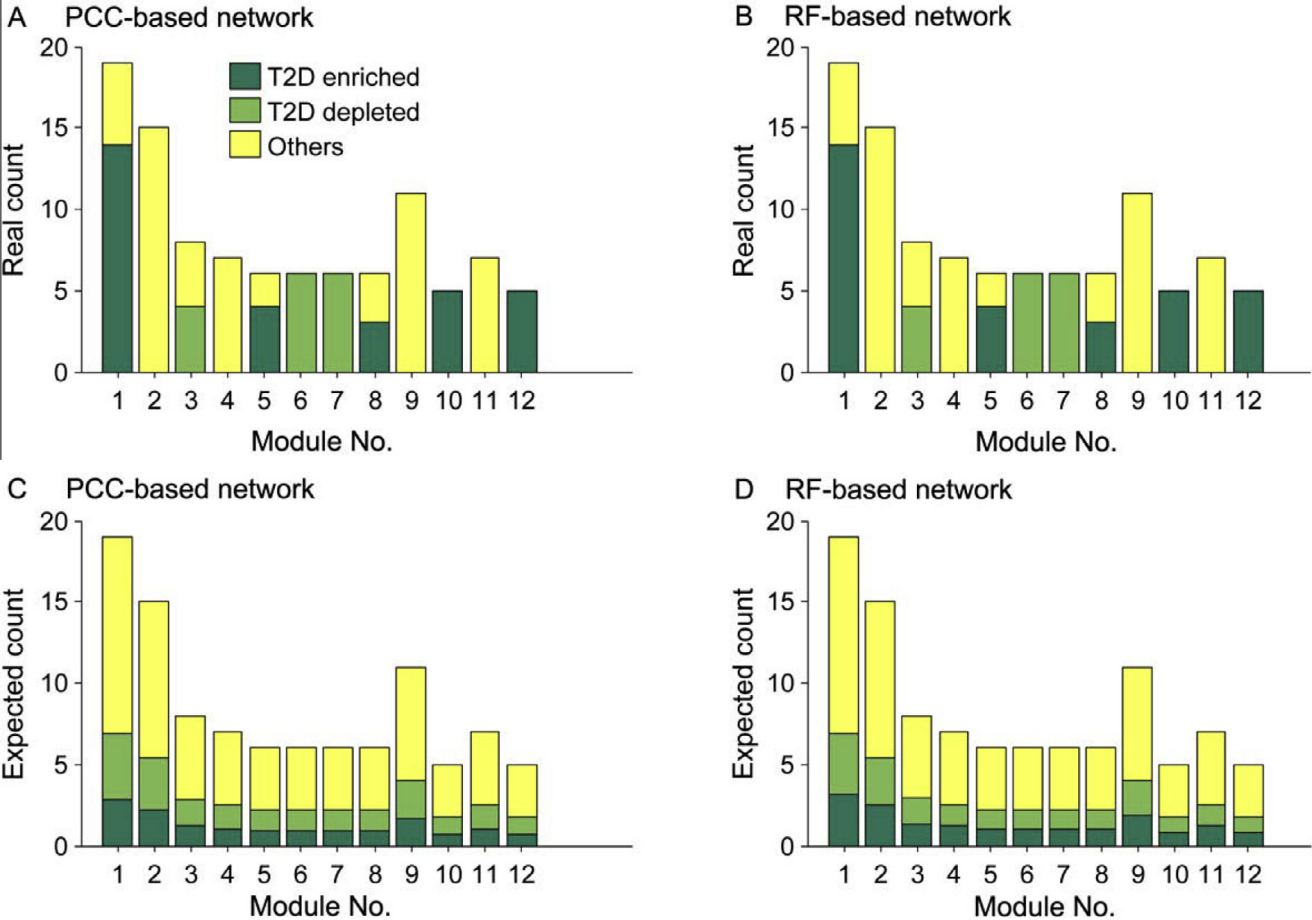
**T2D markers in the common modules of functional networks** Real counts of modules enriched (dark green) and depleted (light green) in T2D markers, together with other functional units (yellow) in the 12 modules in both PCC (**A**) and RF (**B**) networks are shown in the upper row, whereas the corresponding expected counts are presented in panels (**C**) and (**D**), respectively, in the bottom row.

We mapped these KO markers and OG markers to the KO networks and OG networks, respectively to determine how these markers interact with one another (Figure S1) and to illustrate the marker distribution in the two KO networks (Figure 4). As for the node sets of T2D-enriched and T2D-depleted markers in each network, we found that they are significantly densely connected but not randomly distributed (*P* < 10^−4^ for both permutation tests, see Methods). Interestingly, some subsets of particularly highly-connected markers contain the modules described in the previous section. For example, a subset of T2D-depleted KO markers (blue nodes in the red circle in Figure S1) contains Module 1, the largest module that we identified above, which is part of microbial flagellar assembly. Similar results were obtained for the distribution of the T2D-associated OG markers in the OG networks (data not shown).

To explore the relationships between the aforementioned highly-connected markers and network modules, we tested enrichment of these markers in the modules. For the 12 modules commonly found in both KO networks, we counted the number of enriched and depleted markers in the networks based on PCC and RF, and calculated their expected values. Our results showed that actual counts in these two networks were equal and the expected counts were very close too. By Pearson’s χ^2^ test, we found that T2D-enriched markers were statistically significantly enriched in Modules 6 (*P* < 10^−4^) and 7 (*P* < 10^−4^). T2D-depleted markers were significantly enriched in Modules 1 (*P* < 10^−8^), 5 (*P* < 0.003), 10 (*P* < 10^−5^), and 12 (*P* < 10^−5^). Modules 6, 7, and 12 were found to be parts of the microbial transport system. T2D-enriched and T2D-depleted markers that were enriched in the first two and last one module, respectively, might show an association with unbalanced substance transport between microbes and the human intestines. Modules 1, 5, and 10 are parts of the flagellar assembly, chemotaxis proteins [23], and NADH-quinone oxidoreductase [24], respectively. We conjecture that T2D-depleted markers enriched in these three modules may indicate a relationship between diseases and microbial movement or activity.

In further topological analysis, we found that T2D markers that were not present in modules tended to have low degrees. For example, for the PCC-based KO network, we identified two groups of nodes corresponding to T2D-enriched and -depleted markers, respectively, that were not found in the 12 aforementioned modules. For these two groups, we tested whether their average degrees were low by permuting node labels 10,000 times. As a result, the *P* values for T2D-depleted markers and T2D-enriched markers were 0.0006 and 0.0145, respectively, indicating that our finding was statistically significant. Moreover, node stress centrality, which was evaluated by the number of shortest paths passing through a node, was also significantly low (*P* < 10^−4^) for T2D-enriched markers but significantly high (*P* = 0.0227) for T2D-depleted markers. These data partially indicate that the functional T2D markers that were not in modules tended to appear on the network border, which is consistent with previous studies [25]. The aforementioned procedure was also repeated for the RF-based KO network and similar results were obtained (data now shown).

**Supplementary Figure S2:**
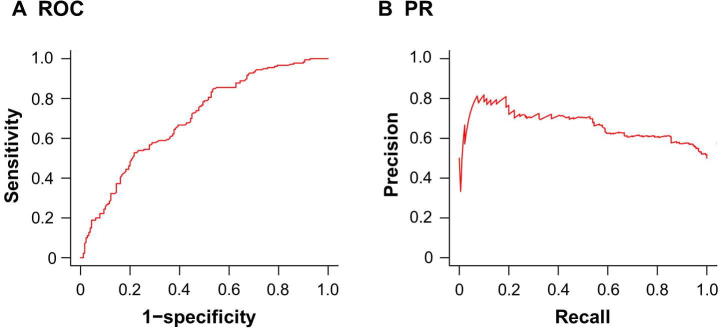
Classification of T2D status based on common modules in the functional networks. ROC (A) and PR curves (B) are plotted to classify the 368 samples. ROC, receiver operating characteristic; PR, precision-recall.

A classification method based on these network markers can be implemented by treating the modules enriched with T2D markers as features. To achieve this goal, we first extracted sample data corresponding to the aforementioned modules. Then, average abundance levels of KOs in each module were calculated as features. For the 183 patient and 185 control samples, we created a classification based on logistic regression with 10-fold cross-validation. Receiver operating characteristic (ROC) and precision–recall (PR) curves are shown in Figure S2. The area under ROC curve (AUC) and area under PR curve (AUPR) are 0.695 and 0.674, respectively, suggesting that the network modules partially reflected the difference between patient and control samples.

### T2D-specific functional networks

As the annotated 6313 KOs of the gut microbiome came from two groups of samples, we further divided it into two separate profiles, one for 183 T2D patients and the other for the 185 healthy individuals, thereby introducing a T2D case–control comparison. Networks were constructed similarly as described above for the general networks. We first removed the uncommon KOs that had zero abundance in some samples and got 2668 and 2870 KOs in case and control profiles of T2D, respectively. Next, we constructed four networks for these two profiles separately by means of PCC and RF.

**Supplementary Figure S3:**
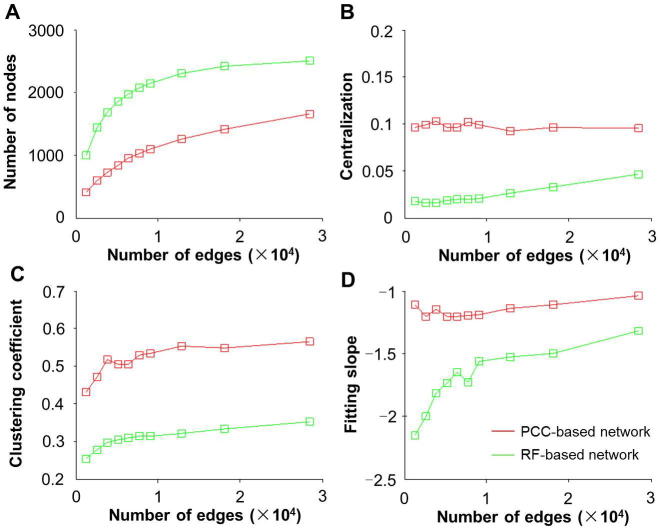
Topological properties of KO networks with different types of threshold. PCC-based and RF-based networks are indicated with red and green lines, respectively.

General topologies of these four networks are shown in Table 2. Obvious topological difference was noticed when using different network construction methods (Figure S3). On the other hand, there is little difference between the networks from case and control samples, indicating that T2D did not affect microbial functional network topology. For further analysis, we compared local structures of these four networks. Similarly, local structures were very similar between case and control networks but different when comparing networks constructed using different methods. Taken all together, we did not observe a significant difference in both general and local structures between the case and control networks.

**Supplementary Figure S4:**
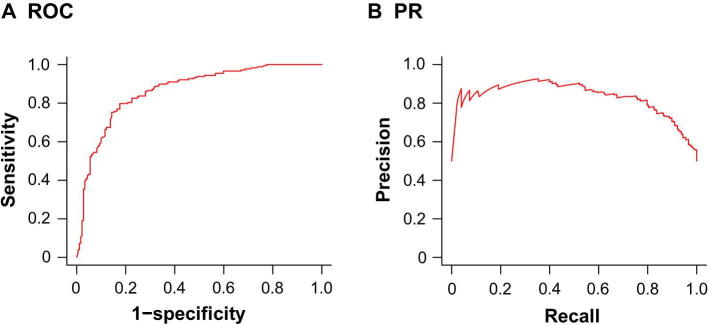
Classification of T2D status based on the modules from control networks. ROC and PR curves are plotted for the classification on the 368 samples, based on all the 7 modules detected in the control network. ROC, receiver operating characteristic; PR, precision–recall.

We found 8 modules shared by both PCC-based and RF-based case networks, and 7 modules shared in both PCC-based and RF-based control networks (Table S2). We then evaluated the abundance difference by the log ratio of the abundance in case network to that in control network. For the case networks, median abundance difference was significantly different from zero in Modules 1, 2, 3, and 5 (all *P* < 0.05). For the control networks, the modules with highly differential abundance were 1, 2, 4, and 6 (all *P* < 0.05). Interestingly, although there is only one T2D marker in Module 6 of the control networks, Module 6 is the only module that shows a significantly high abundance in T2D patients. Thus, we introduced a classifier that uses all the 7 modules in the control networks as features and obtained an AUC value of 0.87, which is higher than that reported previously [10]. The ROC and PR curves of the 10-fold cross-validation experiment are shown in Figure S4.

### Network motifs in functional networks

**Figure 5:**
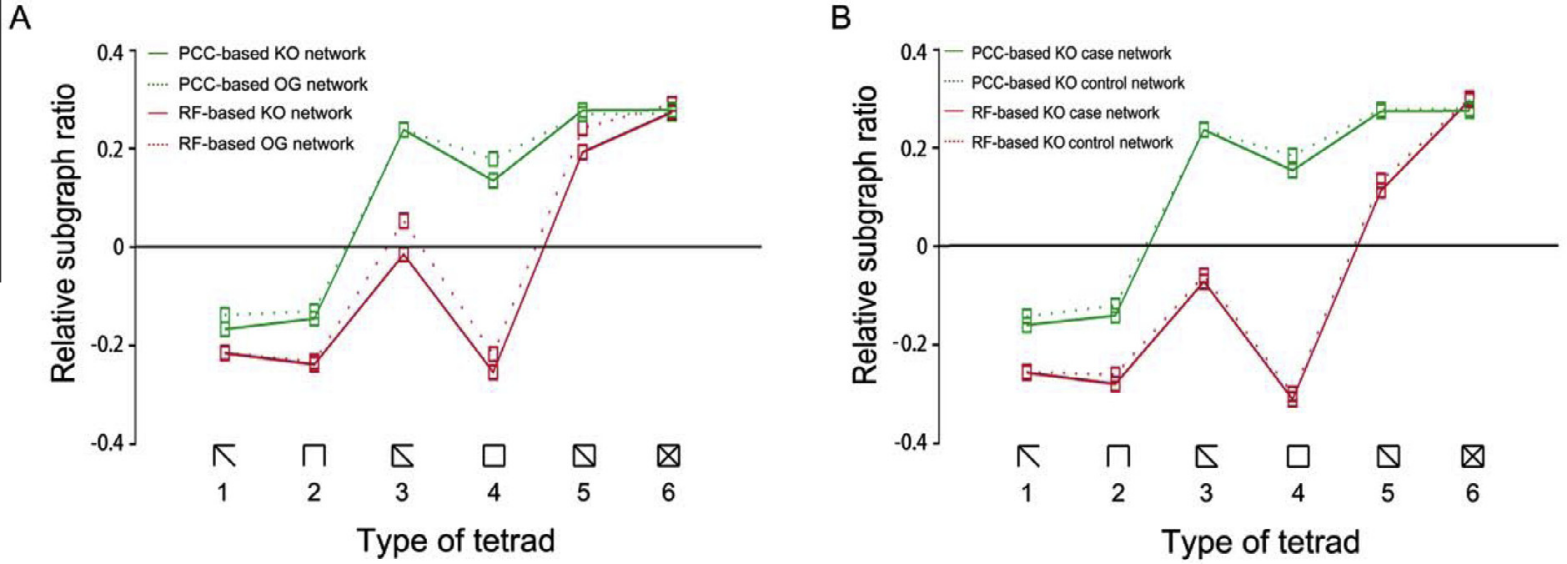
**Network motifs in the functional networks** **A.** The subgraph ratio profile for the functional networks constructed based on PCC (red) and RF (green). Solid line corresponds to KO networks and dash line corresponds to OG networks. **B.** The subgraph ratio profile for the functional networks constructed based on PCC (red) and RF (green) for T2D case and control KO networks. Solid line corresponds to KO case networks and dash line corresponds to KO control networks. For convenience of description, the tetrad types are numbered 1–6 from left to right.

A network motif is a small subgraph of a network that can reflect the local structure of the network. We further analyzed the four networks that were constructed above for motifs. For each network, we counted the numbers of all 6 types of tetrads and compared them to those of random networks by means of mfinder1.2 [26]. Relative significance of the six comparisons was calculated (Figure 5) in order to reduce the influence of the network size. We found that all the 4 networks show a similar trend of motif significance. Networks constructed using the same method had very similar relative motif significance, whereas a large difference in relative motif significance could be detected when comparing networks constructed using different methods. Therefore, difference in relative motif significance between networks is sensitive to methods used for network construction but not to data annotation. This observation indicates that microbial functional networks in the human gut may have the same local structures, while biases could be introduced when employing different methods for network construction. An obvious bias was that PCC method tends to introduce triangles into the network. Accordingly, compared to RF-based networks, we observed fewer Subgraphs 1–5, especially Subgraph 4, in PCC-based networks. Further analysis indicated that local structures of the RF-based networks (data now shown) were more similar to those of the protein structure network constructed based on physical distance [26].

## Conclusions and discussion

In this paper, we propose a framework for constructing and analyzing functional networks of the human gut microbiome. We apply this framework to analysis of a large-scale metagenome sequencing dataset of T2D. We find that the networks constructed using different methods and from different samples may capture different aspects of biological meanings. Our results indicate that PCC method preferentially yields dense modules and triangular local structures when constructing a network, whereas the RF method generates a more scale-free network and detects more protein complexes, suggesting that combinatorial application of both methods would be more efficient.

Our framework can be further extended from the following aspects. First, we normalized the abundance of functional units to the total level of abundance in this study. Although such normalization is widely used in metagenomics studies, it is certainly not the only choice. Methods currently used in the analysis of microarray data, for instance, quantile normalization, should be considered in our future work. Second, we adopted two statistical methods to infer relationships between functional units based on their abundance levels. Besides PCC, there are certainly a lot of alternatives such as Pearson’s correlation combined with mutual information, which is capable of assessing pairwise relationships between two variables. In addition, besides RF, there are also quite a few state-of-the-art machine learning methods ready for use. How to incorporate these methods into our framework is a primary question in our upcoming studies. Finally, although our method is currently focused on the construction and analysis of functional networks in a microbiome, it is also feasible to apply our framework to analysis of 16S rRNA sequencing data, metagenome sequencing data, and metatranscriptome sequencing data. A software package that integrates these extensions into a single program is under development, aiming to facilitate the research in the field of metagenomics.

## Methods

### Metagenome-wide profiling of functional units in the human gut microbiome

We collected high-quality paired-end reads of 368 samples and a human gut microbial gene catalog of 4,267,985 genes annotated with 6313 KOs and 45,684 OGs. KO profiles and OG profiles were obtained using a procedure similar to that described previously [10]. We first mapped these high-quality reads of each sample to the gene catalog by means of a short oligonucleotide alignment tool SOAP2 [27] (options: -m 300 -x 400 -v 2 -n 5). The number of the reads mapped to each gene was counted to calculate the abundance of each gene. We applied the following criterion to implement an alignment as a read count of a gene: both ends of a paired-end read were aligned to the same gene with the proper insert size, or one end of a read was mapped to a gene while the other end of this read was not mapped to the genic region. Let *x_i_* denote the number of reads aligned to gene *i* in a sample, then the relative abundance of gene *i*, denoted as *a_i_*, is calculated as

(1) $$a_{i}=\frac{x_{i}/l_{i}}{\sum_{j=1}^{g}x_{j}/l_{j}}$$

where *l_i_* is the length of gene *i*, and *g* represents the total number of genes. The relative abundance was normalized to the read counts of each gene to eliminate the bias caused by different gene lengths and different sequencing depths across these samples.

With the KEGG and eggNOG annotation of the gene catalog, we calculated the abundance of each KO (or OG) in each sample by summing up the relative abundance levels of all genes annotated with this KO (or OG). Let *b_i_* denote the abundance of the *i*th KO (or OG) in a sample, and the abundance is calculated as

(2) $$b_{i}=\underset{j\in S_{i}}{\sum }a_{j}$$

where *S_i_* is the set of genes annotated by the *i*th KO (or OG), and *a_j_* represents the relative abundance of gene *j*. In this way, we calculated the abundance of 6313 KOs and 45,684 OGs in each of the 368 samples. Finally, we obtained a KO profile and an OG profile by reserving the 2586 KOs and 3320 OGs with nonzero abundance across all samples, which represent the abundance distribution of the common KOs and OGs in the human gut metagenomes. The KO and OG networks can thus be constructed accordingly.

### Construction functional networks for the human gut microbiome

Suppose a profile of the relative abundance contains *N* nodes, the key step for construction of a network is to create an *N* × *N* weight matrix, in which the elements represent the strength of the pairwise association of these *N* nodes. One method for creating the weight matrix is simply to calculate the pairwise correlation coefficients between the abundance distributions of each of the two nodes across all samples. Here, we used PCC between two nodes to weight their association. Let *X_i_* and *Y_i_* denote the abundance of node *X* and *Y* in the *i*th sample; then, PCC is calculated as

(3) $$r=\frac{\sum_{i=1}^{n}(\mathrm{Xi}-\overset{¯}{X})(\mathrm{Yi}-\overset{¯}{Y})}{\sqrt{\sum_{i=1}^{n}{\left(\mathrm{Xi}-\overset{¯}{X}\right)}^{2}}\sqrt{\sum_{i=1}^{n}{\left(\mathrm{Yi}-\overset{¯}{Y}\right)}^{2}}}$$

where *n* is the number of the samples. Pairwise PCCs of all *N* nodes form the *N* × *N* weight matrix. We next assess the statistical significance of the correlation coefficients by a permutation test. Briefly, the significance of association between two nodes is tested by randomly permuting the abundance in all samples, recalculating PCC 10,000 times, and recording the frequency of getting a higher correlation coefficient after permutation than the real one as the *P* value. Then a network can be constructed by setting a *P* value cutoff such as 0.01.

Another way to calculate the weight matrix is based on a machine learning model. With the assumption that the abundance of a node in a sample can be predicted by the abundance of its associated nodes in the same sample, the network construction problem of *N* nodes can be regarded as *N* feature selection subproblems. In each subproblem, the abundance of one node is selected to be the learning target, and the abundance of the remaining *N* − 1 nodes serves as the feature to predict the learning target. Feature selection algorithm is applied to each subproblem to measure the importance of each feature, which is then used as the weight of the association between the target node and the feature node. In this study, we applied the RF algorithm to solve the feature selection subproblems. RF is similar to GENIE3, a state-of-the-art method used to infer gene regulatory networks [28]. In particular, in each subproblem, 1000 decision trees are grown by recursively splitting the samples based on binary tests of the features. A tree node is chosen from randomly-selected $\sqrt{N-1}$ features by optimizing the split to obtain the greatest variance reduction in the learning target. The reduction averaged across all trees can serve as the measure of feature importance. With *N* learning samples, we obtained the importance of all features to all targets, *i.e.*, the pairwise association of all nodes, to form the *N* × *N* weight matrix. We used the R package randomForest to apply this method based on the RF algorithm.

The *N* × *N* weight matrix can be regarded as a fully connected weighted network, and the network construction is accomplished by setting a threshold to filter out the edges with lower weights but keep the edges with higher weights. In most of this study, we completed the networks by keeping 1.5*N* edges with the highest weights, and we call 1.5 as a relative threshold because this threshold yields networks with a better scale-free topology. Different threshold values have also been tested but failed to find significant differences in the identified modules.

### Analysis of functional networks

We used Cytoscape [22] to visualize biological networks, draw the networks, and calculate their topological characteristics such as densities, centralization, diameters, clustering coefficients, and scale-free properties. MCODE [21], a plugin for Cytoscape, was then used to detect modules in biological networks and find the highly-connected modules in the KO and OG networks. To do this, we first intersected the two KO (or OG) networks constructed based on different methods to obtain an intersection KO (or OG) network, and then applied MCODE (with default parameters) to the intersection network to detect modules that would be present in both KO (or OG) networks. Only modules with an MCODE score higher than 2.0 were retained for further analysis.

For a specific node group in the network, we used two hypothesis tests to detect its specific properties. The first one is a permutation test based on shuffling edges in the network. For example, to test whether a node group is densely connected, we first calculate the density of this node group in the network. Then, we switch the edges of this network 10 × *y* times (where *y* is the total number of edges) while keeping the degree of each node unchanged, and thus obtain a randomized network. By calculating densities of the above-mentioned node group in 10,000 such random networks, we obtain an empirical distribution. The empirical *P* value, which means the frequency of greater densities in random networks than in the real network, is finally calculated and used to show the significance of the edge-shuffling permutation test. We used this method to test whether T2D markers are densely connected in functional networks, and whether nodes from a network module are highly connected in other related networks.

The second hypothesis test is also a permutation test which permutes node labels. To determine whether a node group is randomly distributed in a network, we first calculate a topological property such as the density of this node group, and then randomly select the same number of nodes from the network 10,000 times to calculate the empirical *P* value, which is transformed from the frequency of greater values observed. The density, average degree, and average stress centralization are all topological properties of a node group and can be used in this kind of test.

We analyzed the local structures of a network by finding network motifs using mfinder1.2 [26]. For non-directed networks, we used mfinder1.2 to find the numbers of all the 6 tetrads present in the real network and in 100 random networks created by switching edges. For each kind of tetrad, we used its number in the real network, *Nreal*, and its average number in 100 random networks, <*Nrand*>, to calculate the score [26] below:

(4) $$\Delta_{i}=\frac{{\mathrm{Nreal}}_{i}-<{\mathrm{Nrand}}_{i}>}{{\mathrm{Nreal}}_{i}+<{\mathrm{Nrand}}_{i}>+4}$$

After that, the subgraph ratio profile, which is a vector of Δ*_i_* that is normalized to length 1, was calculated, representing a measure of a network’s local structure of a network independent of its size.

## Authors’ contributions

DJ and RJ designed the project. LL and HP performed the experiments. LL, WZ, and HP analyzed the data. LL, MS, and RJ wrote the paper. All authors read and approved the final manuscript

## Competing interests

The authors have declared no competing interests.

## Figures and Tables

**Table 1:** Topological properties of functional networks

**Network property**	**KO network**		**OG network**	
	**PCC-based**	**RF-based**	**PCC-based**	**RF-based**
Nodes	724	1687	906	2118
Edges	3880	3880	4981	4981
Density	0.015	0.003	0.012	0.002
Centralization	0.103	0.016	0.075	0.013
Diameter	20	33	15	27
Clustering coefficient	0.518	0.296	0.507	0.276
Degree distribution fitting slope	−1.147	−1.813	−1.240	−1.922
Degree distribution fitting *R*^2^	0.830	0.925	0.793	0.882

*Note:* KO, KEGG orthologous group; OG, eggNOG orthologous group; PCC, Pearson’s correlation coefficient; RF, random forest.

**Table 2:** Topological properties of T2D-specific functional KO networks

**Network property**	**PCC-based KO network**		**RF-based KO network**		**Intersection**	
	**Case**	**Control**	**Case**	**Control**	**Case**	**Control**
Nodes	648	629	1788	1856	606	606
Edges	4001	4304	4001	4304	999	1247
Density	0.019	0.022	0.003	0.003	0.005	0.007
Diameter	18	12	29	24	20	10
Centralization	0.127	0.125	0.016	0.020	0.031	0.040
Clustering coefficient	0.486	0.497	0.235	0.236	0.302	0.310
Characteristic path length	3.100	3.587	4.475	4.638	4.787	4.116
Degree distribution fitting slope	−0.987	−0.968	−1.910	−1.861	−1.575	−1.536

*Note:* KO, KEGG orthologous group; PCC, Pearson’s correlation coefficient; RF, random forest.

## Supplementary material

Supplementary Figure S1 T2D-associated markers in KO networks and OG networks. (A) PCC-based KO network. (B) RF-based KO network. (C) PCC-based OG network. (D) RF-based OG network. T2D-enriched markers and T2D-depleted markers are indicated by nodes in red and blue, respectively. Modules were detected by using MCODE in Cytoscape. Nodes circled in red represent Module 1, an example module enriched in KEGG pathway for flagella assembly.

Supplementary Table S1 Modules in functional KO networks.

Supplementary Table S2 Modules in T2D-specific functional KO networks.

## References

[1] King A.J., Farrer E.C., Suding K.N., Schmidt S.K. (2012). Co-occurrence patterns of plants and soil bacteria in the high-alpine subnival zone track environmental harshness. Front Microbiol.

[2] Rondon M.R., August P.R., Bettermann A.D., Brady S.F., Grossman T.H., Liles M.R. (2000). Cloning the soil metagenome: a strategy for accessing the genetic and functional diversity of uncultured microorganisms. Appl Environ Microbiol.

[3] Voget S., Leggewie C., Uesbeck A., Raasch C., Jaeger K.E., Streit W.R. (2003). Prospecting for novel biocatalysts in a soil metagenome. Appl Environ Microbiol.

[4] Nautiyal C.S., Chauhan P.S., Nene Y.L. (2007). Medicinal smoke reduces airborne bacteria. J Ethnopharmacol.

[5] Ortiz G., Yague G., Segovia M., Catalan V. (2009). A study of air microbe levels in different areas of a hospital. Curr Microbiol.

[6] Martinez A., Tyson G.W., Delong E.F. (2010). Widespread known and novel phosphonate utilization pathways in marine bacteria revealed by functional screening and metagenomic analyses. Environ Microbiol.

[7] Steele J.A., Countway P.D., Xia L., Vigil P.D., Beman J.M., Kim D.Y. (2011). Marine bacterial, archaeal and protistan association networks reveal ecological linkages. ISME J.

[8] Sunagawa S., Coelho L.P., Chaffron S., Kultima J.R., Labadie K., Salazar G. (2015). Ocean plankton. Structure and function of the global ocean microbiome. Science.

[9] Manichanh C., Rigottier-Gois L., Bonnaud E., Gloux K., Pelletier E., Frangeul L. (2006). Reduced diversity of faecal microbiota in Crohn’s disease revealed by a metagenomic approach. Gut.

[10] Qin J., Li Y., Cai Z., Li S., Zhu J., Zhang F. (2012). A metagenome-wide association study of gut microbiota in type 2 diabetes. Nature.

[11] Qin J., Li R., Raes J., Arumugam M., Burgdorf K.S., Manichanh C. (2010). A human gut microbial gene catalogue established by metagenomic sequencing. Nature.

[12] Cho I., Blaser M.J. (2012). The human microbiome: at the interface of health and disease. Nat Rev Genet.

[13] Zhu C., Jiang R., Chen T. (2015). Constructing a Boolean implication network to study the interactions between environmental factors and OTUs. Quant Biol.

[14] Hurwitz B.L., Westveld A.H., Brum J.R., Sullivan M.B. (2014). Modeling ecological drivers in marine viral communities using comparative metagenomics and network analyses. Proc Natl Acad Sci U S A.

[15] Chaffron S., Rehrauer H., Pernthaler J., von Mering C. (2010). A global network of coexisting microbes from environmental and whole-genome sequence data. Genome Res.

[16] Levy R., Borenstein E. (2013). Metabolic modeling of species interaction in the human microbiome elucidates community-level assembly rules. Proc Natl Acad Sci U S A.

[17] Li B., Yang Y., Ma L., Ju F., Guo F., Tiedje J.M. (2015). Metagenomic and network analysis reveal wide distribution and co-occurrence of environmental antibiotic resistance genes. ISME J.

[18] Du J., Yuan Z., Ma Z., Song J., Xie X., Chen Y. (2014). KEGG-PATH: Kyoto encyclopedia of genes and genomes-based pathway analysis using a path analysis model. Mol Biosyst.

[19] Huerta-Cepas J., Szklarczyk D., Forslund K., Cook H., Heller D., Walter M.C. (2016). eggNOG 4.5: a hierarchical orthology framework with improved functional annotations for eukaryotic, prokaryotic and viral sequences. Nucleic Acids Res.

[20] Albert R., Barabási A.L. (2002). Statistical mechanics of complex networks. Rev Mod Phys.

[21] Bader G.D., Hogue C.W. (2003). An automated method for finding molecular complexes in large protein interaction networks. BMC Bioinformatics.

[22] Smoot M.E., Ono K., Ruscheinski J., Wang P.L., Ideker T. (2011). Cytoscape 2.8: new features for data integration and network visualization. Bioinformatics.

[23] Djordjevic S., Stock A.M. (1998). Structural analysis of bacterial chemotaxis proteins: components of a dynamic signaling system. J Struct Biol.

[24] Brandt U. (2006). Energy converting NADH:quinone oxidoreductase (complex I). Annu Rev Biochem.

[25] Greenblum S., Turnbaugh P.J., Borenstein E. (2012). Metagenomic systems biology of the human gut microbiome reveals topological shifts associated with obesity and inflammatory bowel disease. Proc Natl Acad Sci U S A.

[26] Milo R., Itzkovitz S., Kashtan N., Levitt R., Shen-Orr S., Ayzenshtat I. (2004). Superfamilies of evolved and designed networks. Science.

[27] Li R., Yu C., Li Y., Lam T.W., Yiu S.M., Kristiansen K. (2009). SOAP2: an improved ultrafast tool for short read alignment. Bioinformatics.

[28] Marbach D., Costello J.C., Küffner R., Vega N.M., Prill R.J., Camacho D.M. (2012). Wisdom of crowds for robust gene network inference. Nat Methods.

