# Supplementary figures and images for: Construction and Analysis of Functional Networks in the Gut Microbiome of Type 2 Diabetes Patients
Source: Genomics Proteomics Bioinformatics. 2016 Oct 14;14(5):314–24. doi: 10.1016/j.gpb.2016.02.005 (PMC5093780; doi:10.1016/j.gpb.2016.02.005)

A

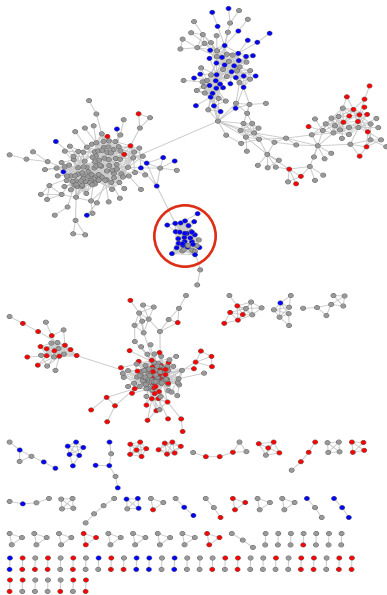

B

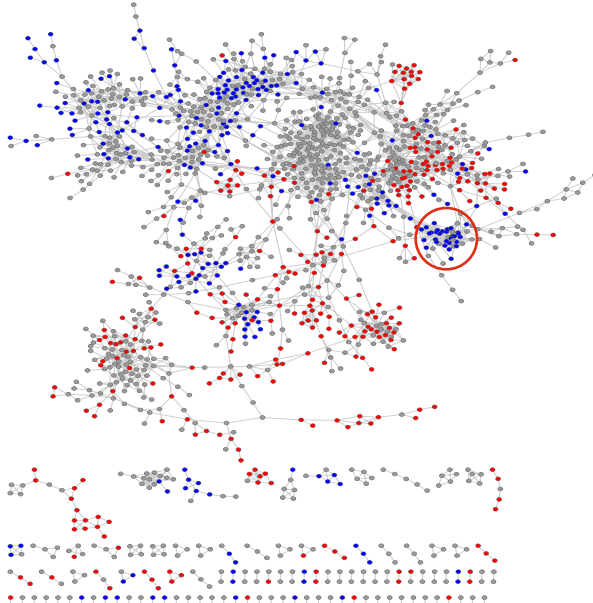

C

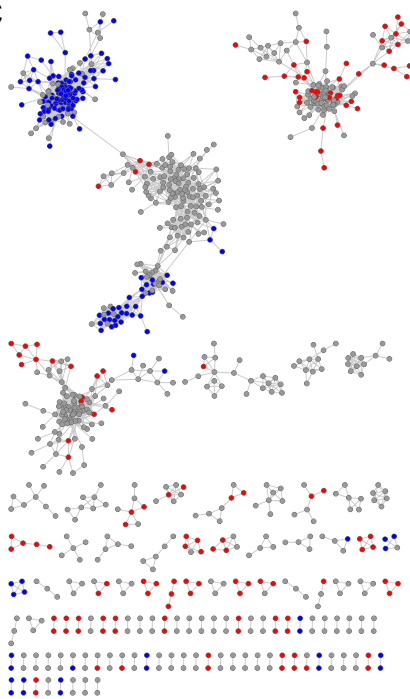

D

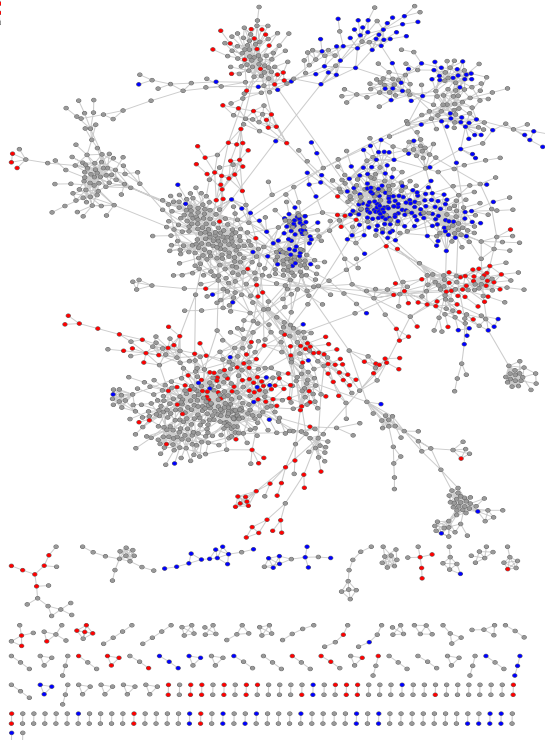

Supplement: Supplementary Figure S1 — T2D-associated markers in KO networks and OG networks. (A) PCC-based KO network. (B) RF-based KO network. (C) PCC-based OG network. (D) RF-based OG network. T2D-enriched markers and T2D-depleted markers are indicated by nodes in red and blue, respectively. Modules were detected by using MCODE in Cytoscape. Nodes circled in red represent Module 1, an example module enriched in KEGG pathway for flagella assembly. [file mmc1.pdf]
